# Obesity Endotypes Unmask Heterogeneous Responses to Healthy Lifestyle Behaviors

**DOI:** 10.64898/2026.08.25.26361367

**Published:** 2026-08-31

**Authors:** Devesh Malik, Min Seo Kim, Injeong Shim, Yang Sui, Roukoz Abou-Karam, Minku Song, Hong-Hee Won, Pradeep Natarajan, Patrick T. Ellinor, Akl C. Fahed

## Abstract

**Background:**

Lifestyle interventions are central to obesity prevention and management, yet interindividual variability in response remains incompletely understood. Here, we leveraged genetically defined, distinct obesity endotypes to examine lifestyle–body mass index (BMI) associations across biological pathways.

**Methods:**

In the UK Biobank, we analyzed 305,713 participants with partitioned polygenic scores (pPSs) representing 10 obesity endotypes. We evaluated interactions between endotype-specific genetic susceptibility and physical activity, diet, sedentary behavior, and sleep on BMI using multivariable linear regression. Primary findings were externally evaluated in the All of Us Research Program using Fitbit-derived lifestyle measures.

**Results:**

Favorable lifestyle behaviors were associated with lower BMI for all obesity endotypes, but the magnitude of these associations varied significantly across endotypes. Higher endotype-specific pPSs strengthened the benefits of physical activity (7 endotypes), healthy diet (3 endotypes), nonsedentary behavior (5 endotypes), and adequate sleep (7 endotypes) on BMI. Distinct endotypes demonstrated the greatest responsiveness to different lifestyle domains, with the metabolically unhealthy endotype showing the strongest interaction with physical activity, metabolically healthy endotype with sedentary behavior, hypothalamic dysregulation endotype with diet, and hypoinsulin 2 endotype with sleep, corresponding to differences in BMI of 0.22–0.49 kg/m² between the highest and lowest pPS deciles. These interaction patterns were consistent in the All of Us cohort.

**Conclusions:**

Obesity endotypes modify the association between lifestyle behaviors and BMI, demonstrating that responsiveness to lifestyle behaviors is heterogeneous and pathway dependent. These findings provide a framework for precision obesity prevention by identifying individuals who may derive greater benefit from specific lifestyle interventions.

## Full Text Availability

The license terms selected by the author(s) for this preprint version do not permit archiving in PMC. The full text is available from the preprint server.

